# Infliximab therapy for intestinal, neurological, and vascular involvement in Behcet disease: Efficacy, safety, and pharmacokinetics in a multicenter, prospective, open-label, single-arm phase 3 study

**DOI:** 10.1097/MD.0000000000003863

**Published:** 2016-06-17

**Authors:** Toshifumi Hibi, Shunsei Hirohata, Hirotoshi Kikuchi, Ukihide Tateishi, Noriko Sato, Kunihiko Ozaki, Kazuoki Kondo, Yoshiaki Ishigatsubo

**Affiliations:** a Center for Advanced IBD Research and Treatment, Kitasato University Kitasato Institute Hospital, Tokyo; b Department of Rheumatology and Infectious Diseases, Kitasato University School of Medicine, Sagamihara; c Department of Internal Medicine, Teikyo University School of Medicine, Tokyo; d Department of Diagnostic Radiology and Nuclear Medicine, Tokyo Medical and Dental University Graduate School of Medicine, Tokyo; e Mitsubishi Tanabe Pharma Corporation, Osaka; f Yokohama City University, Yokohama, Japan.

**Keywords:** infliximab, intestinal Behçet disease, neurological Behçet disease, vascular Behçet disease

## Abstract

Behçet disease (BD) is a multisystem disease associated with a poor prognosis in cases of gastrointestinal, neurological, or vascular involvement. We conducted a multicenter, prospective, open-label, single-arm phase 3 study to determine the efficacy, safety, and pharmacokinetics of infliximab (IFX) in BD patients with these serious complications who had displayed poor response or intolerance to conventional therapy.

IFX at 5 mg/kg was administered to 18 patients (11 intestinal BD, 3 neurological BD [NBD], and 4 vascular BD [VBD]) at weeks 0, 2, and 6 and every 8 weeks thereafter until week 46. In patients who showed inadequate responses to IFX after week 30, the dose was increased to 10 mg/kg. We then calculated the percentage of complete responders according to the predefined criteria depending on the symptoms and results of examinations (ileocolonoscopy, brain magnetic resonance imaging, computed tomography angiography, positron emission tomography, cerebrospinal fluid, or serum inflammatory markers), exploring the percentage of complete responders at week 30 (primary endpoint).

The percentage of complete responders was 61% (11/18) at both weeks 14 and 30 and remained the same until week 54. Intestinal BD patients showed improvement in clinical symptoms along with decrease in C-reactive protein (CRP) levels after week 2. Consistently, scarring or healing of the principal ulcers was found in more than 80% of these patients after week 14. NBD patients showed improvement in clinical symptoms, imaging findings, and cerebrospinal fluid examinations. VBD patients showed improvement in clinical symptoms after week 2 with reductions in CRP levels and erythrocyte sedimentation rate. Imaging findings showed reversal of inflammatory changes in 3 of the 4 VBD patients. Irrespective of the type of BD, all patients achieved improvement in quality of life, leading to the dose reduction or withdrawal of steroids. IFX dose was increased to 10 mg/kg in 3 intestinal BD patients, resulting in the improvement of clinical symptoms, CRP levels, and visual analogue scale score. Safety and pharmacokinetics profiles were comparable to those in patients with rheumatoid arthritis or Crohn disease. These findings support IFX as a new therapeutic option for patients with intestinal BD, NBD, or VBD.

## 1 Introduction

Behçet disease (BD) is a multisystem disease characterized by 4 major symptoms (recurrent oral aphthous ulcers, skin lesions, eye lesions, and genital ulcers) and 5 minor symptoms (arthritis without deformity or ankylosis, epididymitis, gastrointestinal lesions represented by ileocecal ulceration, moderate or severe central nervous system lesions, and vascular lesions).^[1]^ Involvement of the intestinal tract (intestinal BD), the nervous system (neurological BD [NBD]), and the vascular system (vascular BD [VBD]) is rare, although such cases tend to have a poor prognosis.^[2,3]^

Intestinal BD, NBD, and VBD are generally treated using strong immunosuppressive agents such as steroids and immunomodulators. However, these medications are ineffective in some BD patients, who experience repeated relapses, sequelae, and eventually death.^[1,4]^ Further, steroid treatment also poses problems of steroid dependency and adverse drug reactions associated with long-term use. The development of new therapeutic strategies for BD is therefore imperative.

Tumor necrosis factor-α (TNF-α) and interleukin-6 (IL-6) are major inflammatory cytokines involved in the pathogenesis of BD.^[5]^ TNF-α production is elevated in the intestinal tissues of intestinal BD patients^[6]^ and in peripheral blood cells of VBD patients,^[7]^ while IL-6 concentrations are elevated in the cerebrospinal fluid (CSF) of NBD patients.^[8]^

In 2007, infliximab (IFX), an anti-TNF-α monoclonal antibody, was approved in Japan for the treatment of BD-associated refractory retinitis/uveitis, on the basis of the results of a clinical study.^[9]^ Available data on the efficacy of IFX in intestinal BD, NBD, and VBD have been obtained mainly from case studies and retrospective cohort clinical studies,^[10–17]^ and only rarely from prospective clinical studies. Here, therefore, we conducted a multicenter, prospective, open-label, single-arm phase 3 study to evaluate the efficacy, safety, and pharmacokinetics of IFX in BD patients with the serious complications mentioned above. To our knowledge, this is the first prospective multicenter clinical trial of this agent in BD patients with serious complications.

## 2 Methods

This phase 3 clinical study (ClinicalTrials.gov, NCT01532570) was conducted under a prospective, open-label, single-arm clinical design at 21 medical institutions in Japan between January 2012 and May 2014.^[18]^ The protocol was approved by the institutional review board at each medical institution. All patients gave written informed consent. The study was conducted in accordance with the Declaration of Helsinki and Good Clinical Practice. Mitsubishi Tanabe Pharma Corporation sponsored this clinical trial and was responsible for the collection of data.

### 2.1 Patients

The study subjects were patients who had been diagnosed as complete or incomplete type of BD with intestinal BD, NBD, or VBD as per the criteria defined by the Ministry of Health, Labour and Welfare (partially revised in 2010) for Japan, and who had insufficient response or intolerance to conventional therapy. Patients were restricted to those aged 16 to 75 years. With regard to type-specific eligibility criteria, intestinal BD patients had to have intestinal BD-associated symptoms (abdominal pain, diarrhea, melena, etc.) and endoscopic evidence of active ulcers in the intestine. NBD was classified as either acute NBD (ANB) or chronic progressive NBD (CPNB) in accordance with the diagnostic criteria for NBD. ANB patients had to have acute or subacute headache, pyrexia, or focal neurological symptoms and a cell count of ≥6.2 cells/mm^3^ in the CSF or had to have developed acute or subacute symptoms at least twice during the year preceding enrollment, with a cell count of ≥6.2 cells/mm^3^ in the CSF at the onset of symptoms. CPNB patients had to have neuropsychiatric symptoms (dementia-like symptoms, psychiatric symptoms, truncal ataxia, dysarthria, etc.) and a CSF IL-6 concentration of ≥17.0 pg/mL at enrollment and at the most recent measurement within the year preceding enrollment, or a CSF IL-6 concentration of ≥17.0 pg/mL at enrollment and evidence of brainstem atrophy on brain magnetic resonance imaging (MRI). VBD patients had to have active vasculitis lesions (deep vein thrombosis, aortic lesions, etc.) and abnormalities in inflammatory markers such as serum C-reactive protein (CRP) level and erythrocyte sedimentation rate (ESR) at enrollment.

Exclusion criteria were as follows: intestinal manifestations that were not differentiated from acute appendicitis, infectious enteritis, Crohn disease (CD), intestinal tuberculosis, or drug-induced enterocolitis; history of resection of intestinal lesions; neurological manifestations that were not differentiated from infection/allergic meningitis/encephalitis/myelitis, systemic lupus erythematosus, brain/spinal cord tumor, vascular disorders, syphilis, multiple sclerosis, psychiatric disease, or sarcoidosis; vascular manifestations that were not differentiated from Takayasu arteritis, Buerger disease, or arteriosclerotic aneurysm; history of treatment with IFX or other biological drugs for intestinal BD, NBD, or VBD; history of treatment with IFX within 1 year before enrollment for diseases other than intestinal BD, NBD, or VBD or discontinuation of previous IFX treatment due to adverse events; history of a surgical procedure within 4 weeks before enrollment; history or complications of a serious infection requiring hospitalization, opportunistic infection, or tuberculosis within 6 months before enrollment; active hepatitis B or C, or hepatitis B virus carrier status; history of human immunodeficiency virus infection; and a history of congestive heart failure, demyelinating diseases or lymphoproliferative disease, or malignant tumor within 5 years before enrollment.

### 2.2 Study design

IFX at a dose of 5 mg/kg was intravenously infused to patients at weeks 0, 2, and 6, and every 8 weeks thereafter until week 46. If a patient showed a response to IFX by week 30 but thereafter lost the response and the physician deemed that dose escalation was necessary, a dose of 10 mg/kg was administered every 8 weeks until week 46.

Concomitant use of biological drugs, except for IFX, cyclosporin, tacrolimus, and alkylating agents, was prohibited from enrollment to week 54. Changes to the dose of other anti-BD drugs, including azathioprine, 6-mercaptopurine, methotrexate, and aminosalicylic acid, and starting new treatment with or adding these drugs to the regimen, were prohibited from enrollment to evaluation at week 30. However, withdrawal or dose reduction of oral steroids was permitted in line with improvements in symptoms after week 0.

### 2.3 Assessments

Assessments were made in consideration of the specific characteristics of intestinal BD, NBD, and VBD. We assessed clinical symptoms, findings in ileocolonoscopic, brain MRI, computed tomography (CT) angiography, positron emission tomography (PET) studies, CSF (cell count and IL-6 concentration) analysis results, and inflammatory marker (CRP or ESR) levels. We also calculated the percentage of complete responders, defined as those who met the BD type-specific criteria described in Table 1. The primary endpoint was the percentage of complete responders at week 30. Secondary endpoints were the percentages of complete responders at weeks 14 and 54, patient visual analogue scale (VAS) score, Short Form-36 (SF-36) score with regard to physical health, and oral steroid dosage. In addition, we evaluated the major and the other symptoms of BD and assessed the pharmacokinetics and safety of IFX.

**Table 1:**
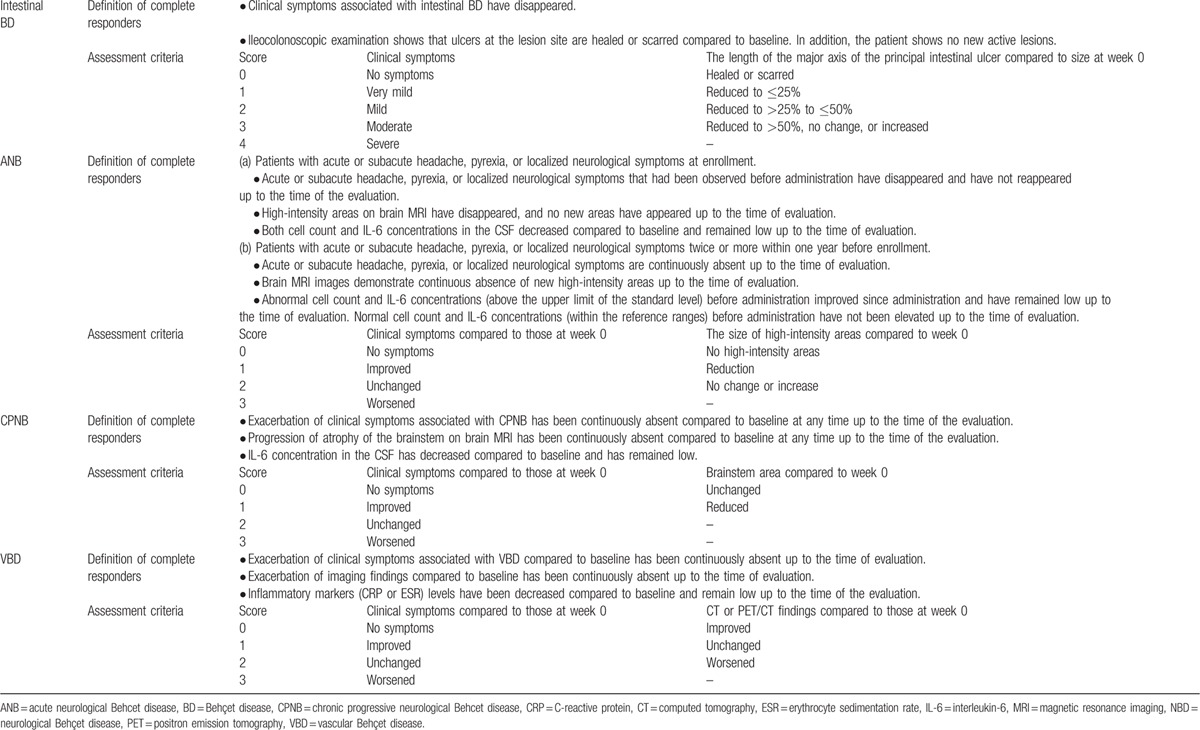
Definition of complete responders and assessment criteria.

For intestinal BD patients, the clinical symptoms were scored at weeks 0, 2, and 6, and every 4 weeks thereafter. Ileocolonoscopy was performed at weeks 0, 14, 30, and 54, and scores were determined in terms of changes in the length of the major axis of the principal intestinal ulcer from that at week 0. Additionally, serum CRP levels were measured at weeks 0, 2, and 6, and every 4 weeks thereafter. For NBD patients, the severity of clinical symptoms was scored at weeks 2 and 6 and every 4 weeks thereafter in terms of changes from the data recorded at week 0. Brain MRI was performed at weeks 0, 14, 30, and 54, and the high-intensity areas in ANB patients and the brainstem area in CPNB patients were scored by comparison to the area measurements made at week 0. Cell count (ANB only) and IL-6 concentrations in the CSF were measured at weeks 0, 14, 30, and 54. Further, for ANB patients, the incidence of acute or subacute attacks occurring between weeks 0 and 30 and between weeks 30 and 54 was determined. For VBD patients, we scored the changes in the degree of swelling, pain, or other VBD-associated clinical symptoms at weeks 2 and 6 and every 4 weeks thereafter in terms of the status at week 0. Changes in CT or PET/CT findings were scored at weeks 14, 30, and 54 by comparison with those at week 0. In addition, the levels of serum CRP and ESR were measured at weeks 0, 2, and 6, and every 4 weeks thereafter, and the incidence of venous thrombosis between weeks 0 and 30 and between weeks 30 and 54 was calculated. Details of the scores are provided in Table 1.

Images obtained from NBD and VBD patients were centrally assessed by 2 BD specialists from the image assessment committee who were blinded to patient symptoms and time of imaging. IFX concentration was measured using an enzyme-linked immunosorbent assay^[19]^ (Mitsubishi Tanabe Pharma Corporation, Osaka, Japan).

### 2.4 Statistical analyses

The full analysis set was used for efficacy analysis. Patient characteristics and efficacy at each time point and at the final point of treatment at 5 mg/kg were assessed in terms of the percentage, frequency, or descriptive statistics. Missing data in the primary endpoint were compensated for using the last observation carried forward method. Safety was assessed on an intention-to-treat basis, and the incidence and percentages of adverse events and adverse drug reactions were calculated.

## 3 Results

### 3.1 Patient disposition

**Figure 1:**
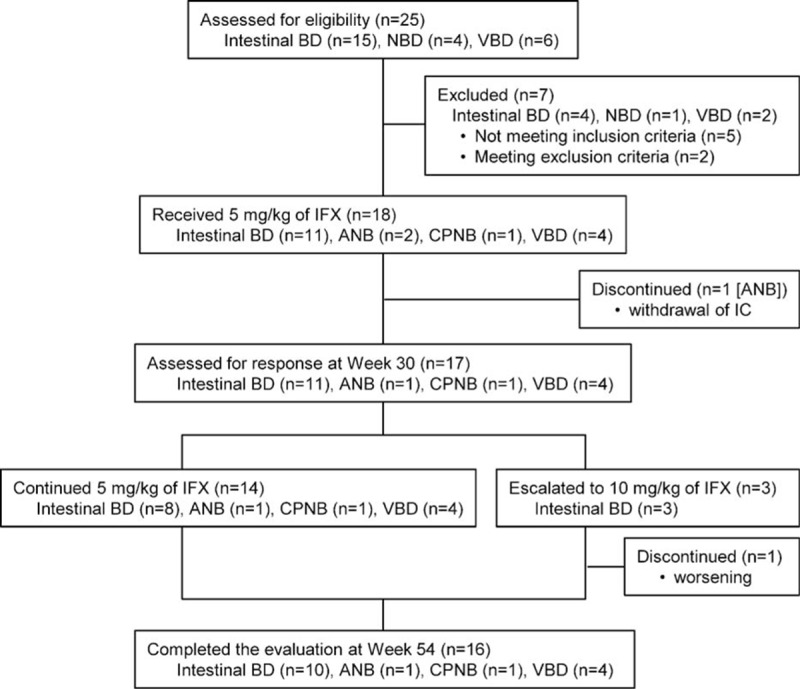
Patient disposition and flow chart of the study. ANB = acute neurological Behçet disease, BD = Behçet disease, CPNB = chronic progressive neurological Behçet disease, IC = informed consent, IFX = infliximab, NBD = neurological Behçet disease, VBD = vascular Behçet disease.

After enrollment, IFX at 5 mg/kg was administered to 18 BD patients (11 intestinal BD, 3 NBD [2 ANB and 1 CPNB], and 4 VBD) (Fig. 1). Before week 30, 1 ANB patient withdrew consent and discontinued treatment with IFX. Subsequently, 14 BD patients (8 intestinal BD, 2 NBD, and 4 VBD) received a dose of 5 mg/kg IFX until week 54. The dose was increased to 10 mg/kg after week 30 in 3 intestinal BD patients, 1 of whom then discontinued treatment due to exacerbation of symptoms.

Patient characteristics are shown in Table 2. Skin lesions were the most common major symptom (15/18 [83%]). Among the 11 intestinal BD patients, the ileum (9 patients; 82%) was the most common lesion site, followed by the cecum and ascending colon (3 patients each; 27%), transverse colon and descending colon (1 patient each; 9%), and others (2 patients; 18%). No patients had lesions in the rectum. Of the 4 VBD patients, 3 (75%) had venous lesions (deep vein thrombosis of the lower extremity, 1; others, 2), and 1 (25%) had arterial lesions (arterial occlusion).

**Table 2:**
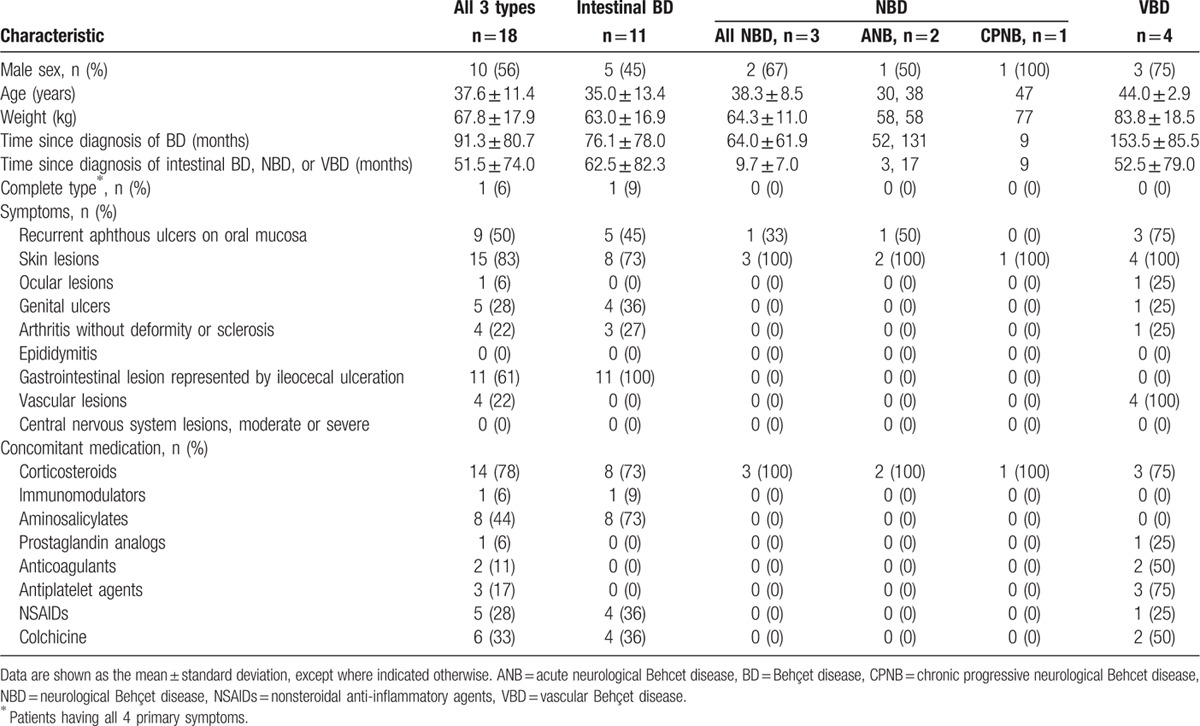
Baseline characteristics of patients.

### 3.2 Efficacy

Eleven (61%) of the 18 BD patients showed a complete response at an early stage (week 14). This ratio of complete responses was maintained at week 30, the primary endpoint (61%, 11/18) (Table 3), and then increased to 69% (11/16) at week 54 and 67% (12/18) at the final point of 5 mg/kg therapy. Efficacy of IFX was maintained for up to 1 year. By BD type, the percentage of complete responders at week 30 was 55% (6/11) among intestinal BD patients, 33% (1/3) among NBD patients, and 100% (4/4) among VBD patients.

**Table 3:**
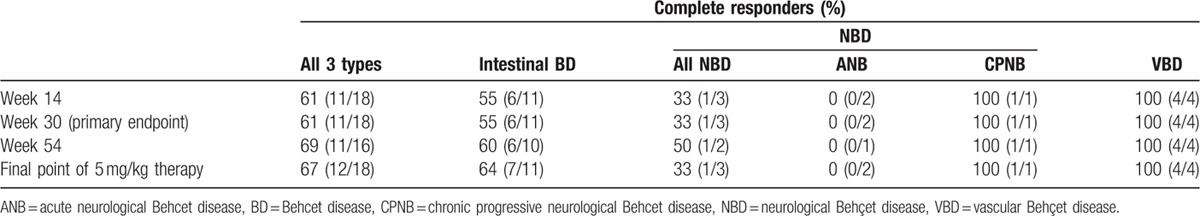
Proportion of complete responders.

### 3.3 Intestinal BD

**Figure 2:**
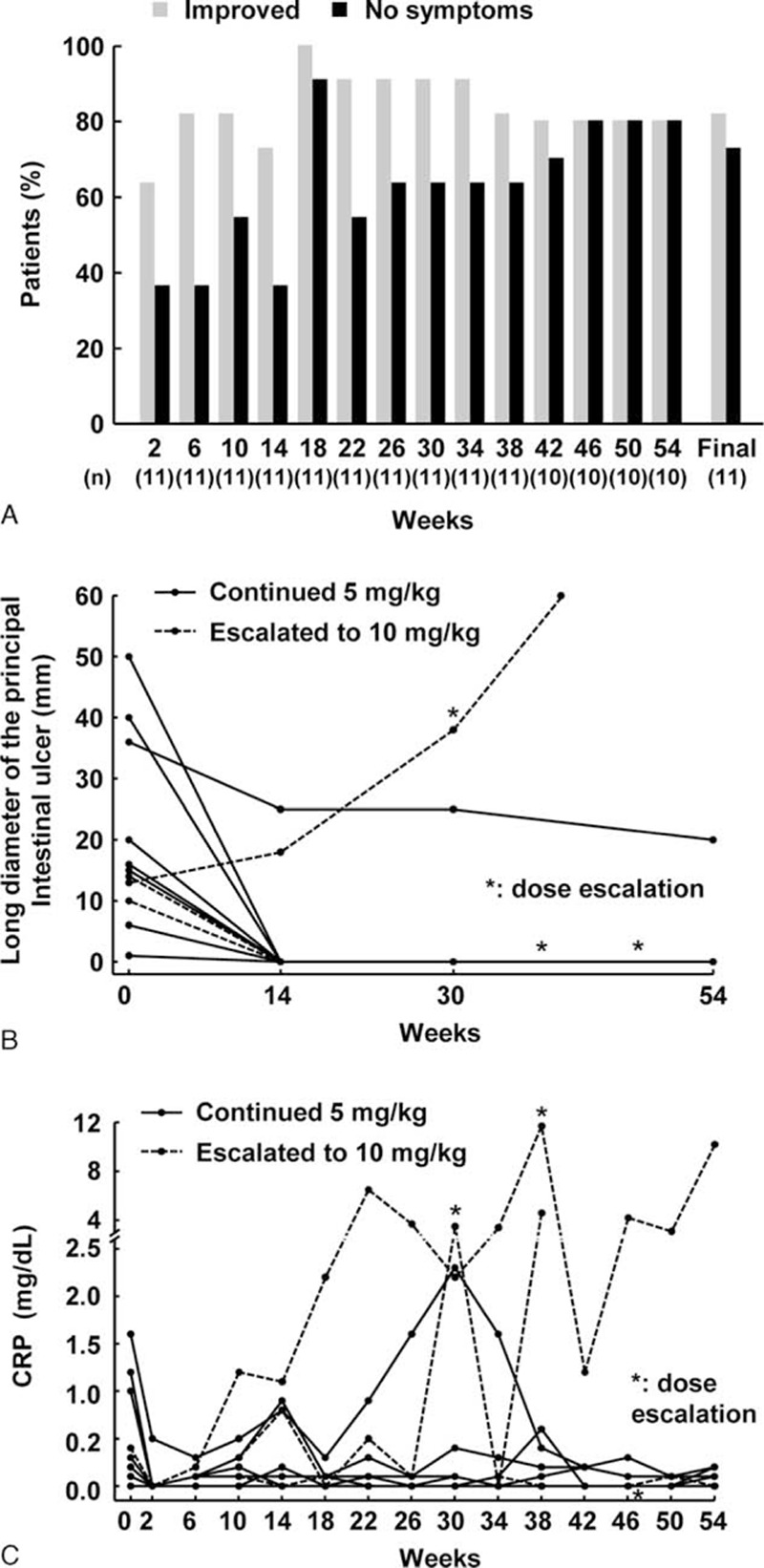
Efficacy of infliximab on intestinal Behçet disease. (A) Percentage with improved or cured clinical symptoms, (B) changes in size of major ulcers for each patient, and (C) changes in serum C-reactive protein (CRP) levels for each patient.

Clinical symptoms at week 0 were very mild in 3 patients with intestinal BD, mild in 5, and moderately severe in 3. The percentage of patients showing an improvement in clinical symptoms compared to that at week 0 was 64% (7/11) at week 2 and gradually increased to 73% (8/11) at week 14 and 91% (10/11) at week 30 (Fig. 2A). In addition, the percentage of patients showing an improvement in clinical symptoms was 80% (8/10) at week 54 and 82% (9/11) at the final point of 5 mg/kg therapy. The percentage of patients with no clinical symptoms was 36% (4/11) at week 2, which gradually increased to 64% (7/11) at week 30 and 80% (8/10) at week 54. The percentage of intestinal BD patients with no clinical symptoms at the final point of 5 mg/kg therapy was 73% (8/11).

Healing or scarring of the principal intestinal ulcer was recorded at week 14 in 9 (82%) patients, and most patients showed no recurrence until week 54 (Fig. 2B). The percentage of patients with healing or scarring of the principal ulcer was 82% (9/11) at week 30, 89% (8/9) at week 54, and 82% (9/11) at the final point of 5 mg/kg therapy.

Median serum CRP level was 0.20 mg/dL (interquartile range: 0.00–1.00 mg/dL) at week 0. This then decreased or remained low from week 2 up to week 54 in most intestinal BD patients (Fig. 2C), as follows: 0.00 mg/dL (0.00–0.00 mg/dL) at week 2, 0.15 mg/dL (0.00–0.80 mg/dL) at week 14, 0.10 mg/dL (0.00–2.20 mg/dL) at week 30, 0.10 mg/dL (0.00–0.20 mg/dL) at week 54, and 0.10 mg/dL (0.00–0.20 mg/dL) at the final point of 5 mg/kg therapy.

IFX dose was increased to 10 mg/kg after week 30 in 3 patients with intestinal BD due to loss of response. Among these, the dose was increased in 1 patient at week 46 due to recurrence of abdominal pain, which caused some difficulties in daily activities. After dose escalation, the clinical symptoms and VAS score of the patient improved. In the 2nd patient, IFX dose was increased at week 38 due to abdominal pain, fever, diarrhea, and melena with high serum CRP level. However, although serum CRP levels, VAS score, and clinical symptoms improved after dose escalation, the disease could not be controlled and worsened at week 54. In the 3rd patient, IFX dose was increased at week 30 due to recurrence of abdominal pain and an increase in the size of the principal ulcer. Although the stomach pain, serum CRP level, and VAS score improved after dose escalation, the disease worsened after 2 doses of 10 mg/kg, and the patient was withdrawn from the study.

### 3.4 NBD

**Figure 3:**
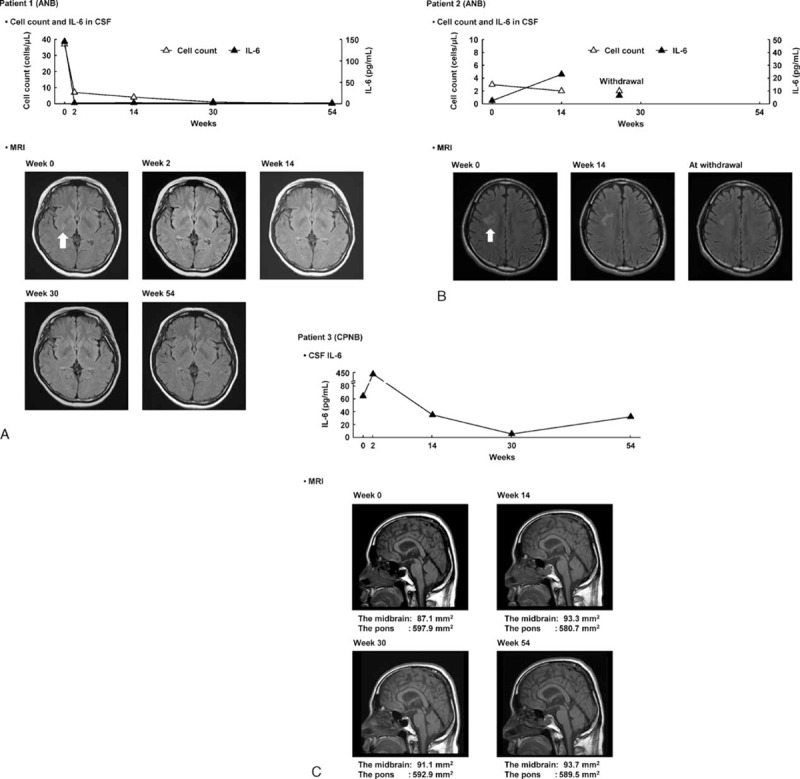
Efficacy of infliximab on neurological Behçet disease. (A) CSF cell count, CSF IL-6 concentration, and FLAIR MRI images in patient 1 (ANB); (B) CSF cell count, CSF IL-6 concentration, and FLAIR MRI images in patient 2 (ANB); and (C) CSF IL-6 concentration and T1-weighted MRI images in patient 3 (CPNB). ANB = acute neurological Behçet disease, CPNB = chronic progressive neurological Behçet disease, CSF = cerebrospinal fluid, IL-6 = interleukin-6, MRI = magnetic resonance imaging.

One ANB patient (patient 1) had acute symptoms (fever and headache) and elevated cell count and IL-6 concentration in the CSF at week 0 (37 cells/μL and 145 pg/mL, respectively) (Fig. 3A). At week 2, the acute symptoms disappeared, and the cell count and IL-6 concentration decreased to 7 cells/μL and 1.8 pg/mL, respectively. Aside from a mild headache at week 22, no acute symptoms occurred at other measurement time points, and CSF analysis did not show any evidence of inflammation until week 54 (at week 54: cell count, ≤1 cell/μL; IL-6 concentration, 1.5 pg/mL). FLAIR MRI at week 0 showed high signal intensity in the posterior part of the right lenticular nucleus to the posterior limb of the internal capsule, while FLAIR MRI images showed a small area of high-intensity in the genu of the right internal capsule to the globus pallidus; however, these areas shrank in size by weeks 2, 14, and 30, and the high-intensity area in the right internal capsule was further reduced at week 54. Although 2 episodes of attack (acute symptoms) were reported during the 12 months before the start of administration (1st episode: diplopia and aphagia; 2nd episode: vertigo, nausea, fever, headache, and diplopia), only a single mild attack (mild headache) occurred after the start of IFX treatment. MRI did not depict any new areas of high signal intensity during the study period.

The other ANB patient (patient 2) reported headache as a chronic symptom but did not have any acute symptoms or abnormalities on CSF analysis (cell count 3 cells/μL and IL-6 concentration of 2.5 pg/mL) at week 0 (Fig. 3B). Although the patient experienced headache and dull headache as chronic symptoms until week 22, no acute symptoms occurred after the start of IFX treatment. However, this patient withdrew consent and discontinued the study at week 22. CSF cell count was 2 cells/μL both at week 14 and withdrawal. CSF IL-6 concentration was 23.0 pg/mL at week 14 and 6.5 pg/mL at withdrawal. The high-intensity areas in the right forehead and parietal lobe seen on FLAIR MRI of the head at week 0 were reduced at both week 14 and at withdrawal. Although this patient experienced 2 episodes of attack (acute symptoms) in the year preceding the start of IFX treatment (1st episode: headache; 2nd episode: headache and malaise), the attacks did not occur after the start of treatment, although the evaluation period was relatively short.

One CPNB patient (patient 3) showed slightly slow reactions with a relatively high CSF IL-6 concentration of 64.5 pg/mL at week 0 (Fig. 3C). Despite an increase to 430.0 pg/mL at week 2, no changes in clinical symptoms compared to week 0 were noted. At week 6, all clinical symptoms had resolved, and no symptoms (0 points) appeared until week 54. CSF IL-6 concentration declined to 35.1 pg/mL at week 14 and further decreased to 5.4 pg/mL at week 30, but slightly increased at week 54 (32.1 pg/mL). At week 0, T1-weighted MRI of the head did not reveal any abnormalities. The areas of the midbrain and pons were 87.1 and 597.9 mm^2^, respectively, at week 0 and remained nearly unchanged until week 54 (93.7 and 589.5 mm^2^, respectively).

### 3.5 VBD

**Figure 4:**
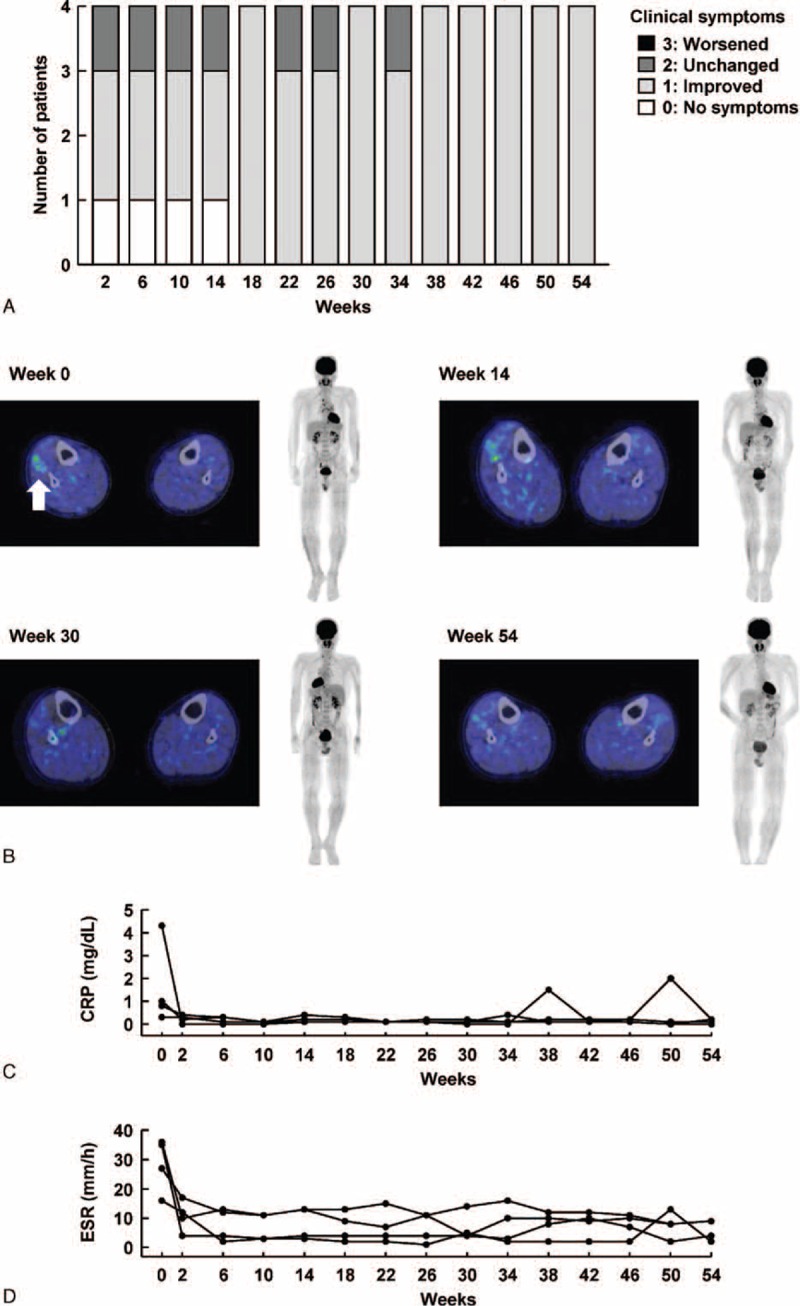
Efficacy of infliximab on vascular Behçet disease. (A) Change in clinical symptoms, (B) PET/CT images of thrombophlebitis of the lower legs in a representative patient, (C) changes in serum CRP level for each patient, and (D) changes in serum ESR for each patient. CRP = C-reactive protein, CT = computed tomography, ESR = erythrocyte sedimentation rate, PET = positron emission tomography.

At week 2, clinical symptoms were improved (1 point) or absent (0 points) in 3 of the 4 VBD patients (Fig. 4A). Aggravation of clinical symptoms did not occur in any of the VBD patients, and improvement was maintained between weeks 38 and 54. Imaging findings at week 14 showed improvement in inflammation (0 points) in 3 of the 4 patients, which continued until week 54. In the remaining VBD patient, imaging findings depicted no aggravation until week 54. PET/CT images of the patient with thrombophlebitis of the lower legs who showed improvement are shown in Fig. 4B. At week 0, inflammation was observed around the veins of the lower legs (slightly more severe in the right lower leg). These inflammatory changes ameliorated at week 14 and showed further improvement at weeks 30 and 54. At this last evaluation, both lower limbs showed no marked difference in severity. With respect to the patient with arterial occlusion in the right armpit, the right radial artery was not palpable and the right extremity was numb at week 0. The arterial occlusion slightly improved at week 14 and further improved at weeks 30 and 54.

Changes in the serum CRP level and ESR in VBD patients are shown in Fig. 4C and D. The median serum CRP level was 0.90 mg/dL at week 0, which decreased to 0.25 mg/dL at week 2. Levels stayed low at weeks 14, 30, and 54, with median values of 0.15, 0.10, and 0.15 mg/dL, respectively. Median serum ESR was 31.0 mm/hour at week 0, declining to 11.0, 8.5, 4.5, and 6.5 mm/hour at weeks 2, 14, 30, and 54, respectively. Neither clinical symptoms nor imaging findings showed evidence of the development of new lesions throughout the investigational period. No newly occurring venous thrombosis was noted in any of the 4 VBD patients during the study period.

### 3.6 Improvement of quality of life (QOL) in patients with gastrointestinal lesions, central nervous system lesions, and vascular lesions

**Figure 5:**
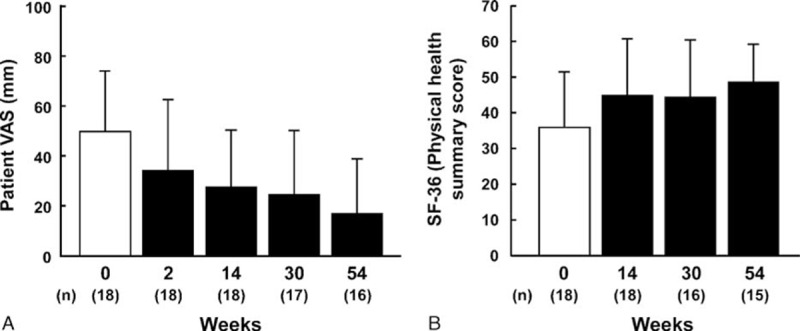
Effects of infliximab on quality of life in patients with intestinal, neurological, and vascular Behçet disease. (A) Patient VAS score, (B) SF-36 score (physical health summary score). Data are shown as the means ± standard deviation. SF-36 = Short Form-36, VAS = visual analogue scale.

Among BD patients, VAS score (mean ± standard deviation) improved over time, from 49.8 ± 24.2 mm at week 0 to 34.2 ± 28.3 mm, 27.5 ± 22.9 mm, 24.6 ± 25.5 mm, and 17.0 ± 21.8 mm at weeks 2, 14, 30, and 54, respectively (Fig. 5A). The SF-36 score (mean ± standard deviation) was 35.9 ± 15.6 at week 0, and then improved to 44.9 ± 15.9, 44.4 ± 16.0, and 48.6 ± 10.6 at weeks 14, 30, and 54, respectively (Fig. 5B). VAS and SF-36 scores improved across all types of BD.

### 3.7 Steroid dose reduction and withdrawal

Of the 14 BD patients treated with oral steroids at week 0, doses were reduced in 6 at week 14 and in these 6 as well as in an additional 1 at week 30 (n = 7 total; Table 4). Two of these 7 patients were eventually free from treatment with steroids, both with intestinal BD. These 7 BD patients remained on reduced steroid doses until week 54 or were free from steroid use by then. By week 54, steroid doses were reduced in another 2 patients, and one of these 2 patients eventually withdrew from steroid use. Steroid dose was reduced across all types of BD. Six of the 9 patients with reduced steroid doses showed complete responses, and 2 of these 6 were free from steroid treatment successfully. No patients required initiation of oral steroid treatment or an increase in dosage during the study period.

**Table 4:**
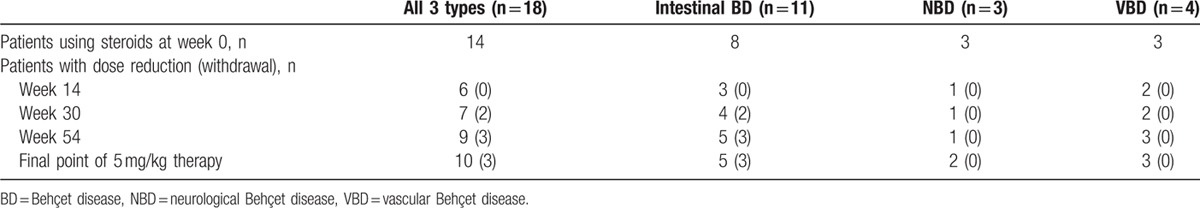
Incidence of dose reduction and withdrawal of steroids.

### 3.8 Effects on major symptoms of BD

**Figure 6:**
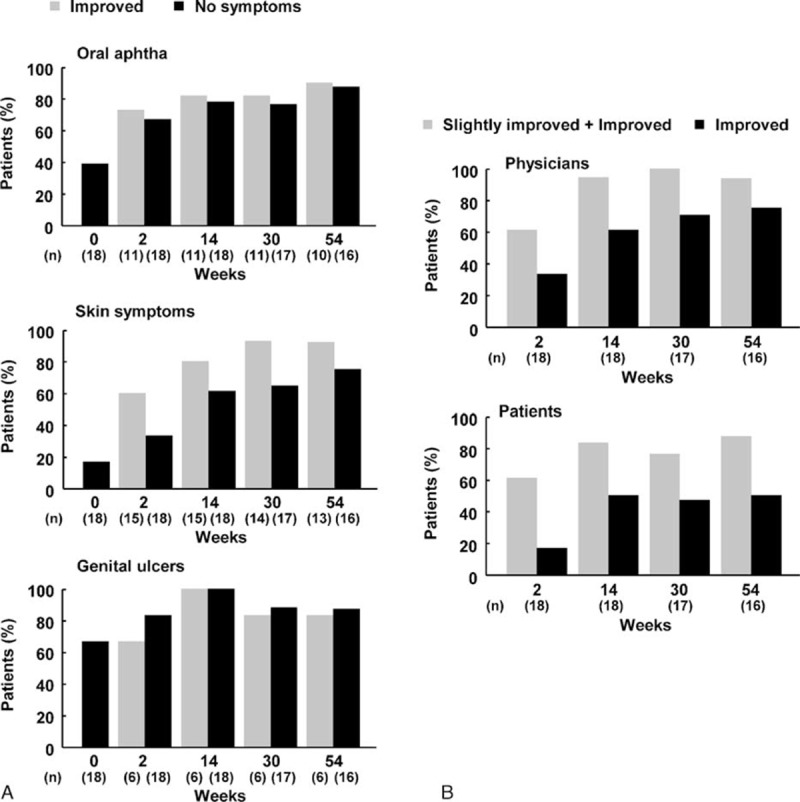
Efficacy of infliximab on major symptoms of Behçet disease (BD) and global assessment of overall disease activities by physicians and patients. (A) Major symptoms of BD (oral aphtha, skin symptoms, and pudendal ulcer), (B) assessment of overall disease activities of BD (assessed by physicians or patients).

More than 60% of BD patients who had oral aphtha (n = 18), skin symptoms (n = 18), or genital ulcers (n = 18) at week 0 had improved by week 2 (Fig. 6A). The percentage showing improvement after week 6 ranged from 73% to 91% for oral aphtha, 73% to 100% for skin symptoms, and 83% to 100% for genital ulcer and the percentage with no symptoms (0 points) increased after week 2, suggesting that the efficacy of IFX was maintained. One patient had eye symptoms at week 0 that resolved by week 2 and did not recur during the course of the study.

### 3.9 Effects on all symptoms of BD (assessed by physicians or patients)

At week 2, the percentages of patients showing slight improvement (1 point) or improvement (0 points) in all symptoms of BD as assessed by physicians and patients were 61% (11/18) and 61% (11/18), respectively (Fig. 6B). Percentages of patients showing slight improvement or improvement as assessed by the physicians and patients were 94% (17/18) and 83% (15/18) at week 14, 100% (17/17) and 76% (13/17) at week 30, and 94% (15/16) and 88% (14/16) at week 54, respectively. These percentages clearly remained high, irrespective of whether reporting was by physicians or patients. Percentages of patients showing improvement as assessed by physicians were 33% (6/18) at week 2, 61% (11/18) at week 14, 71% (12/17) at week 30, and 75% (12/16) at week 54; the corresponding percentages as assessed by patients were 17% (3/18), 50% (9/18), 47% (8/17), and 50% (8/16).

### 3.10 Pharmacokinetics

**Figure 7:**
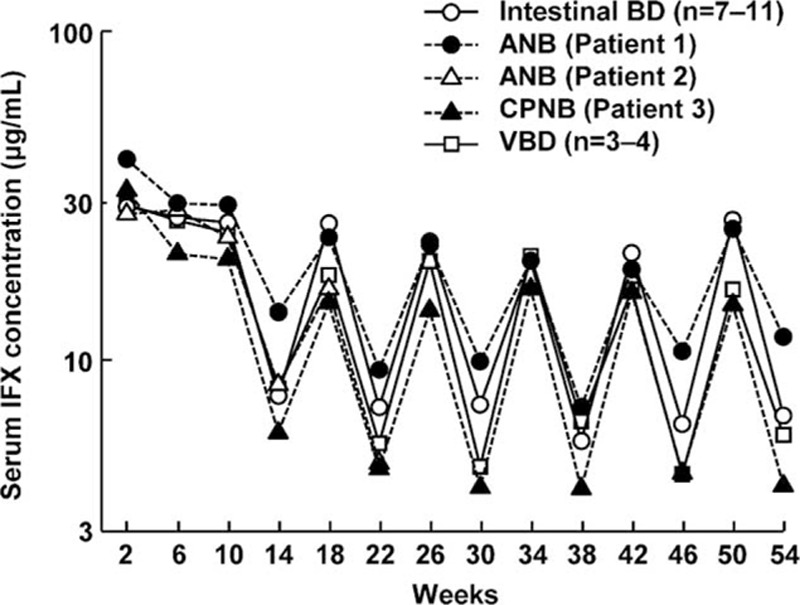
Changes in serum infliximab concentration. Data of patients with intestinal and vascular BD are shown as the median. ANB = acute neurological Behçet disease, BD = Behcet disease, CPNB = chronic progressive neurological Behçet disease, IFX = infliximab, VBD = vascular BD.

No marked differences in serum IFX concentration were noted by BD type among patients treated with a dose of 5 mg/kg. Trough serum IFX concentrations were stably maintained for each BD type after week 14 (Fig. 7). In the 3 intestinal BD patients whose doses were increased to 10 mg/kg after week 30 due to loss of response, trough serum IFX concentrations rose after the dose escalation (7.78–18.74, 0.96–6.01, and 4.39–9.77 μg/mL).

### 3.11 Safety

The incidence of adverse events across all patients was 94% (17/18), with the following type breakdown: 91% (10/11) in intestinal BD patients, 100% (3/3) in NBD patients, and 100% (4/4) in VBD patients, showing no marked difference among BD types (Table 5). Infections occurred in 11 patients. Regarding the infections reported in more than 10% of patients, we observed upper respiratory tract infection (5/18), nasopharyngitis (4/18), gastroenteritis (2/18), and infectious enteritis (2/18). With regard to serious adverse events, worsening of the underlying disease and cataracts were observed in 1 intestinal BD patient. A causal relationship between these serious adverse events and IFX was ruled out because the 1st patient had similar worsening of the underlying disease with small bowel perforation even before enrollment, and the cataract in the 2nd patient was considered to be a result from a long-term use of oral steroids. In the 3 intestinal BD patients who were administered an increased dose of 10 mg/kg, the only adverse event that occurred after the dose escalation was the worsening of the underlying disease, as mentioned above.

**Table 5:**
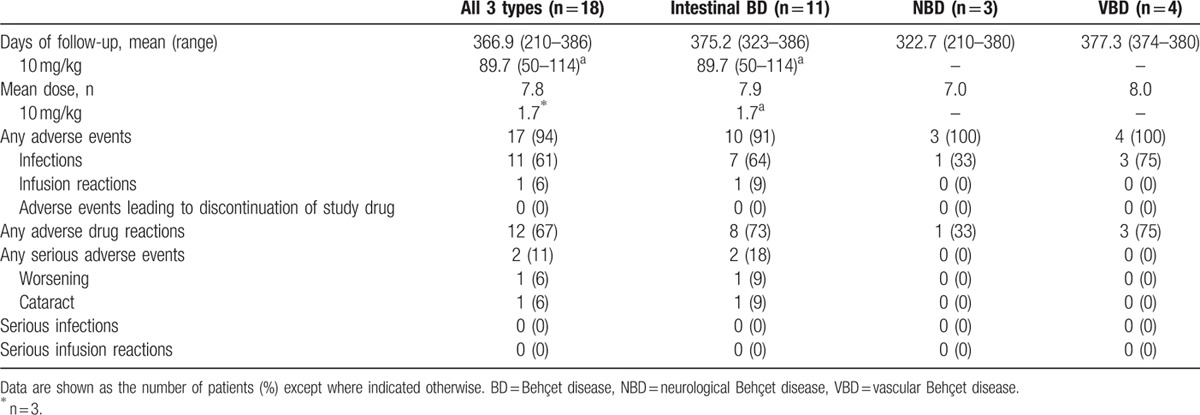
Safety profile.

## 4 Discussion

In those patients with intestinal BD, NBD, and VBD who had displayed a poor response or intolerance to conventional therapy, efficacy of IFX was observed soon after starting treatment and was maintained until week 54 of treatment. No major differences in the safety or pharmacokinetics of IFX were noted between our study population versus patients receiving treatment with IFX in rheumatoid arthritis (RA) or CD.

IFX has been reported to exert its effects in BD via a number of mechanisms, including neutralization of TNF-α and subsequent suppression of γδT cell expansion, activation, and cytotoxic activity^[20]^ and a decrease in the levels of CSF IL-6 through cytotoxic effects on monocytes/macrophages.^[15]^ Further, IFX has been found to be effective in treating intestinal BD,^[10–12,17]^ NBD,^[13–15,17]^ and VBD.^[16,17]^ However, most of these reports were case studies and retrospective cohort clinical studies, and little data are available from prospective clinical studies.

We obtained novel data on IFX through comprehensive assessment of clinical symptoms, morphological characteristics, and levels of inflammatory markers in intestinal BD, NBD, and VBD patients. Approximately 60% of our population treated with IFX showed a complete response at week 14, and efficacy was maintained until the end of the study at week 54. The percentage of complete responders at week 30 (primary endpoint) was 61%. Similar to the French retrospective study,^[17]^ the findings from our prospective study suggest that IFX is indeed effective for BD patients with these serious complications.

In intestinal BD patients, the clinical symptoms and endoscopic findings improved soon after starting treatment, as evidenced by reductions in serum CRP levels, and this efficacy was maintained until week 54. Our present findings for IFX were comparable to previous findings from studies using other TNF inhibitors, such as adalimumab^[21]^ or etanercept,^[22]^ in terms of efficacy against intestinal BD. Healing or scarring of the principal ulcer was achieved in more than 80% of such patients in the present study, suggesting that IFX has potent mucosal healing effects. Mucosal healing in intestinal BD patients has been reported to predict the long-term prognosis, in terms of the risk of clinical relapse and surgery.^[23]^ Therefore, IFX is expected to maintain low disease activity in the long term, which subsequently may reduce the need for surgery.

In CPNB patients, IFX has been reported to reduce CSF IL-6 concentrations and thereby inhibit the progression of CPNB.^[15]^ In the present study as well, IFX lowered CSF IL-6 concentrations and resolved clinical symptoms in a CPNB patient without the occurrence of brainstem atrophy. In ANB patients, IFX lowered the cell count and IL-6 concentrations in the CSF and inhibited the onset of attacks. Onset of attacks in NBD patients may lead to the onset or progression of cerebellar disorder-associated gait disorders, dysarthria, and dysuria and then to cognitive impairment, neurological disorders, and personality changes.^[24]^ Although our study involved a relatively small number of NBD patients, our findings suggest that IFX inhibits the onset of attacks and improves or inhibits NBD-associated progressive pathological conditions. Therefore, IFX may improve the overall prognosis in NBD patients.

In VBD patients, analysis of clinical symptoms, imaging findings, and inflammatory markers demonstrated the efficacy of IFX soon after starting treatment, with effectiveness maintained until week 54. PET/CT findings showed that IFX administration improved thrombophlebitis and arterial occlusion by decreasing the severity of inflammation. Neither the aggravation of VBD nor inflammation was newly seen in any patient receiving IFX treatment during the present study, suggesting that the antiinflammatory effects of IFX inhibit the progression of VBD.

QOL is relatively poor among BD patients.^[25]^ However, in the present study, IFX improved the VAS and SF-36 scores, irrespective of the type of BD. This effect suggests that, by resolving BD symptoms, IFX improves the QOL of BD patients. In the present study, IFX treatment also allowed a reduction in dose or withdrawal of steroid treatment in all BD types, while also improving BD-associated symptoms. IFX improved not only the major symptoms, including recurrent oral aphthous ulcers, skin lesions, eye lesions, and genital ulcers, but also all the other symptoms of BD, from treatment initiation up to week 54. One patient with eye symptoms at baseline did not develop any eye symptoms or ocular attacks throughout the course of the study. In addition, IFX has been reported to suppress the frequency of ocular attacks in patients with BD with refractory uveoretinis.^[9]^ We therefore believe that IFX treatment is effective against all symptoms of BD.

The efficacy of IFX in treating RA and CD has been reported to depend on its serum concentration, and dose escalation up to 10 mg/kg is possible.^[26–31]^ In the present study, 3 intestinal BD patients who met the dose escalation criteria were administered IFX at 10 mg/kg. After dose escalation, clinical symptoms, serum CRP level, and VAS score continuously or temporarily improved, accompanied by an increase in serum IFX concentration. In spite of the paucity of a number of BD patients, our observations suggest that a dose of 10 mg/kg is useful in those who lose response at 5 mg/kg, as is the case with RA and CD.

No marked differences in safety were noted among patients by BD type. The safety of IFX administration in the present study was comparable to that determined previously in studies with other diseases, including RA,^[26,27]^ CD,^[28–31]^ and BD with refractory uveoretinitis.^[9]^

Interpretation of our study is limited by the small population and a lack of any controls. BD is a rare disease in Japan, particularly intestinal BD, NBD, and VBD. After consulting with the Pharmaceuticals and Medical Devices Agency of Japan, the sample size was set to be at least 3 patients per disease type, with a total of 15 patients, in consideration of the number of these patients available and the registration criteria for this study. Our study population was therefore quite small. In addition, we were unable to include a control group for ethical reasons as well as a lack of appropriate control agents and the small number of subjects. We therefore consider it necessary to collect additional data using other methods, such as postmarketing surveillance.

In conclusion, IFX is effective and well tolerated in the treatment of intestinal BD, NBD, and VBD patients with poor response or intolerance to conventional therapy. We believe that the antiinflammatory effect of IFX at the sites of intestinal, neurological, and vascular lesions may help improve morphological changes (ileocecal ulcerations, high-intensity lesions on brain, brainstem atrophy, thrombophlebitis, etc.), clinical symptoms, and QOL and also help reduce steroid dose. IFX may therefore represent a promising new therapeutic option for use in BD patients with these serious conditions.
